# Metastatic ER+ Breast Cancer: Mechanisms of Resistance and Future Therapeutic Approaches

**DOI:** 10.3390/ijms242216198

**Published:** 2023-11-11

**Authors:** Farah Raheem, Suganya Arunachalam Karikalan, Felipe Batalini, Aya El Masry, Lida Mina

**Affiliations:** 1 Mayo Clinic, Phoenix, AZ 85054, USA; raheem.farah@mayo.edu (F.R.);; 2 Phoenix Country Day School, Paradise Valley, AZ 85253, USA

**Keywords:** endocrine resistance, ER+ breast cancer, ESR1 mutations, PIK3CA mutations, PI3K/Akt/mTOR pathway, MAPK pathway, PROTAC, ShERPA, SERCAs, AURKA inhibitors

## Abstract

Endocrine therapy is the main treatment for hormone receptor-positive (HR+) breast cancer. However, advanced tumors develop resistance to endocrine therapy, rendering it ineffective as the disease progresses. There are several molecular mechanisms of primary and secondary endocrine resistance. Resistance can develop due to either alteration of the estrogen receptor pathway (e.g., ESR1 mutations) or upstream growth factors signaling pathways (e.g., PI3K/Akt/mTOR pathway). Despite progress in the development of molecularly targeted anticancer therapies, the emergence of resistance remains a major limitation and an area of unmet need. In this article, we review the mechanisms of acquired endocrine resistance in HR+ advanced breast cancer and discuss current and future investigational therapeutic approaches.

## 1. Introduction

Breast cancer is the second leading cause of death among women in the United States. In 2023, it is estimated that 300,590 women will be diagnosed with breast cancer and 43,700 will die from the disease [1]. Breast cancer treatment is challenging and highly dependent on the biology of the tumor. Approximately 70% of breast cancer cases are estrogen receptor (ER) positive and are treated with endocrine therapy. However, not all endocrine-driven tumors are created equal. The primary molecular targets of endocrine therapy have been the ER and the progesterone receptors (PR). In breast cancers expressing ER/PR, the receptor–ligand interaction promotes the development and proliferation of cancer cells [2]. Although endocrine therapy is considered the most effective and well-tolerated targeted treatment, strategies have moved from a one size fits all to a more molecularly targeted approach due to breast cancer heterogeneity.

**Figure 1:**
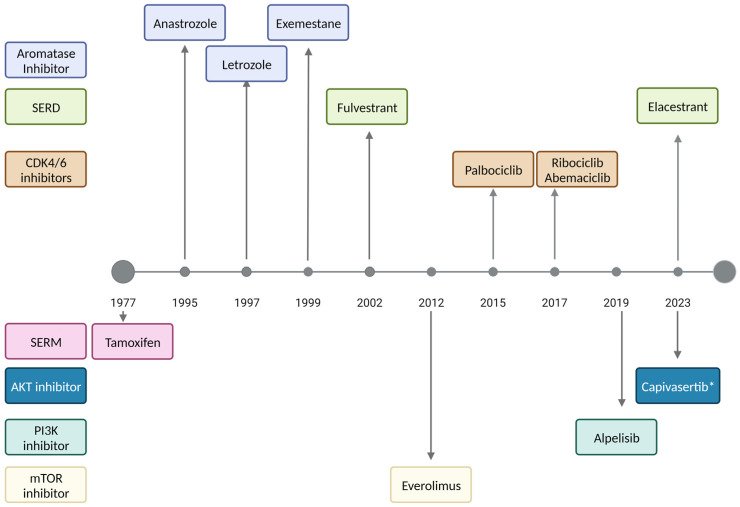
Current endocrine and targeted treatment of metastatic ER+ breast cancer. Antiestrogen therapy including selective estrogen receptor modulators (tamoxifen), aromatase inhibitors (anastrazole, letrozole, exemestane), and selective estrogen receptor degraders (fulvestrant) continues to be the treatment backbone of hormone receptor-positive breast cancer since tamoxifen FDA approval in 1977. Improvement in the understanding of the molecular biology of estrogen-positive breast cancer led to the development of effective targeted therapies such as CDK4/6 inhibitors (palbociclib, ribociclib, abemaciclib) and inhibitors of PI3K/Akt/mTOR signaling pathway (alpelisib, capivasertib), as well as improvement in the delivery and potency of traditional endocrine therapy including oral selective estrogen receptor degraders (elacestrant). * Anticipated regulatory approval during the fourth quarter of 2023. Abbreviations: AKT, AKT Serine/Threonine Kinase 1; CDK, cyclin-dependent kinase; ER, estrogen receptor; mTOR, mammalian target of rapamycin; PI3K, phosphoinositide 3-kinases; SERD, selective estrogen receptor degraders; SERM, selective estrogen receptor modulator.

Despite advances in cancer treatments, the development of resistance remains a major challenge, especially among women with metastatic disease. The standard first-line treatment for HR+ metastatic breast cancer (MBC) includes endocrine therapy with or without CDK4/6 inhibitors (Figure 1). Recently, we witnessed the introduction of additional targeted therapies. PIK3CA inhibitors, mTOR inhibitors, PARP inhibitors, and novel oral selective estrogen receptor degraders (SERDs) are now being used beyond first-line therapy. Other treatment options for recurrent endocrine-resistant ER+ MBC include toxic chemotherapy with limited efficacy. In this review, we summarize the mechanisms of acquired endocrine resistance in HR+ MBC and discuss therapies currently in use and under investigation.

## 2. Endocrine Resistance

Endocrine therapy (ET) is the main treatment for HR+ breast cancer. However, advanced tumors develop resistance to ET rendering it ineffective as the disease progresses [3]. There are several molecular mechanisms of primary and secondary endocrine resistance described in the literature [4]. Crosstalk between ER and other signaling pathways such as survival signaling pathway, growth factor receptors pathway, and other signal transduction pathways have been implicated in primary and acquired resistance to endocrine therapy. In general, endocrine resistance can develop due to either alteration of the estrogen receptor pathway (e.g., ESR1 mutations) or upstream growth factors signaling pathways (e.g., PI3K/Akt/mTOR pathway). Although we witnessed progress in the development of novel, molecularly targeted anticancer treatments in breast cancer, the optimal combination and sequencing of such therapies to overcome endocrine resistance remain a challenge.

Primary endocrine resistance is defined as disease recurrence within 2 years of adjuvant endocrine treatment or progression during the first 6 months of first-line ET for advanced or MBC. Secondary or acquired resistance is defined as disease recurrence after the first two years of adjuvant ET, within 12 months of completing adjuvant ET or progression 6 months after initiating ET for MBC [5].

**Figure 2:**
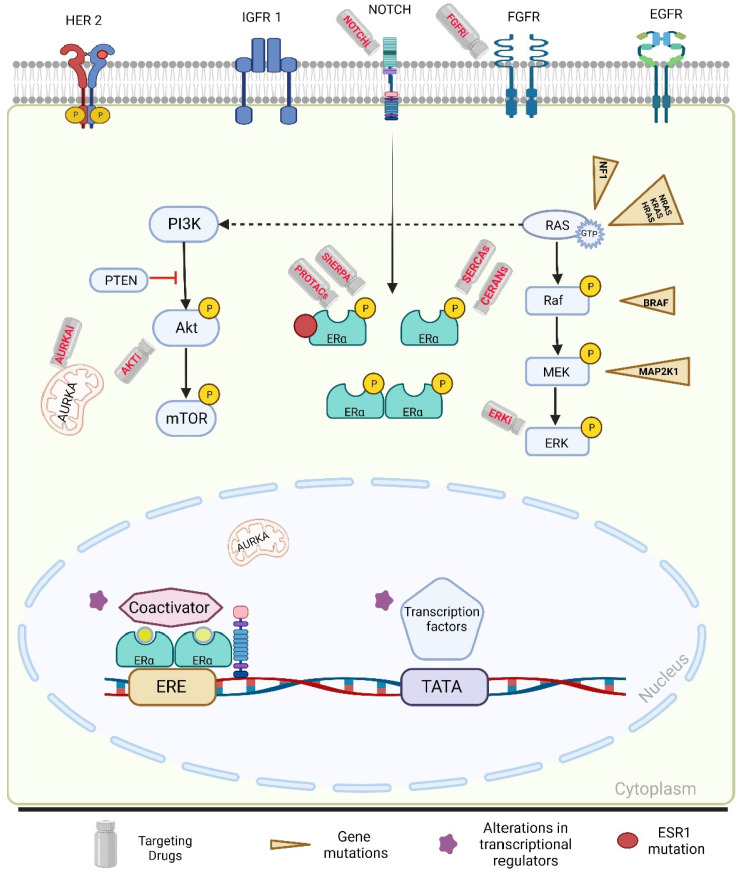
Common mechanisms of secondary endocrine resistance in advanced ER+ breast cancer. Aberrant activation of RTKs (HER2, IGFR1, FGFR, EGFR) by mutations or amplifications leads to activation of the PI3K and MAPK signaling pathways. NF1 loss-of-function mutations result in constitutively active RAS leading to downstream activation of both PI3K and MAPK pathways. PI3K and MAPK pathways activation induces ER phosphorylation and ligand-independent activation leading to transcription of ER-regulated genes despite estrogen blockade with endocrine therapy. Targeting upstream activation of RTKs (FGFR inhibition) or downstream oncogenic proteins of the PI3K pathway (AKT inhibition) and MAPK pathway (ERK inhibition) provides therapeutic options that can potentially overcome secondary endocrine resistance when combined with antiestrogen therapy. Point mutations of ER (ESR1 mutations) are another common mechanism of endocrine resistance rendering aromatase inhibitors ineffective. Oral SERDs ± targeted therapies present as an effective treatment option in ESR1 mutated tumors. CERANs, SERCAs, ShERPAs, and PROTACs are novel agents with therapeutic potential to overcome resistance to traditional endocrine therapies and are under development. NOTCH signaling upregulation has been shown to promote breast cancer stem-like cell expansion and its pharmacologic disruption can lead to reversal of resistance to antiestrogen therapy. AURKA overactivation has been linked to downregulation of estrogen receptors leading to endocrine resistance and worse prognosis. Novel agents targeting AURKA are being investigated as a potential therapeutic approach to overcome endocrine resistance. Abbreviations: AURKA, aurora kinase A; BRAF, v-raf murine sarcoma viral oncogene homolog B1; CERAN, Complete Estrogen Receptor Antagonist; EGFR, epidermal growth factor receptor; ERK, extracellular signal-regulated kinase; ER, estrogen receptor; ERE, estrogen receptor element; FGFR, fibroblast growth factor receptor; GTP, Guanosine-5’-triphosphate; HRAS, Hras proto-oncogene; HER, human epidermal growth factors receptor; IGFR, insulin-like growth factor; KRAS, Kirsten rat sarcoma virus; mTOR, mammalian target of rapamycin; MAP2K, mitogen-activated protein kinase; MEK, mitogen-activated protein kinase kinase; NOTCH, notch signaling pathway; NF, nuclear factor; NRAS, neuroblastoma RAS viral oncogene Humalog; PROTAC, Proteolysis Targeting Chimera; PI3K, phosphoinositide 3-kinases; PTEN, phosphatase and TENsin homolog; RAS rat sarcoma signaling; Raf, rapidly accelerated fibrosarcoma; ShERPA, selective human estrogen receptor partial agonist; SERCA, selective estrogen receptor covalent antagonist.

While the term endocrine resistance traditionally refers to resistance to estrogen blockade, we use the term generally to refer to resistance to suppressing ER and we focus on mechanisms of secondary resistance in this review. Common mechanisms of secondary resistance are depicted in Figure 2.

### 2.1. Mechanisms of Primary Endocrine Resistance

One mechanism of primary endocrine resistance is loss of ER expression [6]. Conversion from ER+ to ER-negative breast cancer is reported in 10% to 20% of cases and highlights the importance of retesting receptors upon disease progression [7,8]. Proposed mechanisms of loss of ER expression include epigenetic changes (histone deacetylation and methylation of ER promoter), hypoxia, overexpression of human epidermal growth factor receptor-2 (HER2), and mitogen-activated protein kinase (MAPK) pathway activation with the latter being associated with transcriptional repression of ER gene resulting in endocrine resistance [9]. In contrast to secondary resistance, mutations in the ER alpha gene (ESR1) are rare and only found in <1% of treatment-naïve breast tumors [10].

### 2.2. Mechanisms of Secondary Endocrine Resistance

#### 2.2.1. ESR1 Mutations

ESR1 gene encodes for ERα. Mutations in the ESR1 gene were first described in 1997 but their role in endocrine resistance was not established until 2013 due to the historical practice of mainly sequencing treatment-naïve breast tumors [11]. The identification of ESR1 mutations is most common among patients with metastatic disease treated with aromatase inhibitors (AI), occurring in 20% to 40% of patients with MBC [12]. Among patients with recurrent breast cancer after treatment with adjuvant AI, ESR1 mutations are found in 4–5% of cases [13,14,15]. Second-line ET monotherapy for MBC is associated with reduced clinical benefits with median progression-free survival (PFS) of 2–6 months compared to 1–4 years when used in the first-line setting suggesting the presence of ESR1 mutation, which can predict resistance to AI monotherapy [16]. This highlights the importance of sequencing metastatic and advanced breast tumors upon progression to identify acquired molecular aberrations.

Clinically, ESR1 mutations predict poor response to AI, as shown in the SoFEA and EFECT trials comparing fulvestrant to exemestane for ER+ MBC after progression on nonsteroidal AI [13,17]. Patients with ESR1 mutations vs. those with ESR1 wild type (WT) at enrollment had shorter PFS (2.4 vs. 4.8 months) and 1-year overall survival (OS) rate of 62% vs. 79%, respectively. ESR1 mutations tend to develop 3 to 6 months before radiologic progression on AI [18,19], and typically occur in the ligand binding domain (LBD) of ERα leading to constitutively active ER that becomes independent of estrogen, and therefore is not affected by estrogen depletion (Figure 2) [20,21]. The constitutively active form of ER due to ESR1 mutation is more stable in the active confirmation with decreased susceptibility to proteolytic degradation [21]. Common activating ESR1 point mutations within the LBD are D538G and Y537S. Others include Y537N, Y537C, L536H, L536P, L536R, S463P, and E380Q [22,23,24,25]. ESR1 fusions are less common but can lead to the absence of LBD and complete resistance to ET agents that target the LBD such as SERMs (e.g., tamoxifen) and SERDs (e.g., fulvestrant) [26,27]. Different ESR1 mutations may confer different levels of resistance as suggested by evidence that the Y537S mutation leads to greater resistance to estrogen depletion, SERMs and SERDs compared to the D538G mutation [11].

In culture and xenograft models, ESR1 mutants were shown to have 30-fold and 40-fold decreased binding affinity to tamoxifen and fulvestrant, respectively, and required higher doses to inhibit transactivation and cell proliferation function [20,21,28]. However, resistance to tamoxifen and fulvestrant may not be completely explained by ESR1 mutations alone. Furthermore, ESR1 mutation is not enriched in patients progressing on tamoxifen treatment for MBC [24] and the association between ESR1 mutation and resistance to tamoxifen has not been proven outside of in vitro studies [11]. As a result, novel SERMS present a treatment option to overcome endocrine resistance driven by ESR1 mutation, and there are agents currently in development such as lasofoxifene [29,30].

While in vitro studies demonstrated decreased binding affinity of fulvestrant to mutated ESR1, clinical evidence of complete resistance to fulvestrant in the presence of activating ESR1 mutations in the LBD has been conflicting, and it is unknown whether higher doses of fulvestrant would overcome resistance [11]. In the SoFEA and EFECT trials discussed above, patients with ESR1 mutations derived clinical benefits from fulvestrant [17]. In the PALOMA-3 trial in which patients with HR+ MBC who progressed on prior AI were randomized to fulvestrant with or without palbociclib, there was a numerically lower PFS among patients with ESR1-mutant tumor compared to those with ESR1 WT (3.6 vs. 5.4 months) in the fulvestrant arm, but statistical significance was not reached [13].

ESR1 mutation alone cannot fully explain endocrine resistance when MBC is treated with a combination of ET plus targeted treatments such as inhibitors of CDK4/6, PIK3CA, or mTOR. While ESR1 mutation is a common mechanism of acquired endocrine resistance in ER+ MBC, other identified mechanisms of resistance include alterations in the PI3K/Akt/mTOR, RAS-MAPK, and CDK4/6-Rb-E2F pathways, and ESR1 loss, amplification, and translocation [31].

#### 2.2.2. PI3K-Akt-mTORC1 Pathway

The oncogenic phosphatidylinositol 3-kinase (PI3K)/Akt pathway is a major activating pathway implicated in tumorigenesis, cancer cell survival, and development of resistance [32]. Aberrant PI3K/Akt pathway activation in malignancy can occur due to somatic mutations in PIK3CA, phosphatase and tensin homolog (PTEN), Akt, TSC1, and mechanistic target of rapamycin (mTOR) genes [33]. In HR+, HER2 negative MBC, overactivation of the PI3K/Akt pathway is common and occurs in approximately 50% of cases [34]. The rates of PI3KCA activating mutations in HR+ MBC with primary and secondary endocrine resistance are similar [31], which, in keeping with the fact that these are clonal alterations and suggests that the development of subclonal PIK3CA mutations is not a common response to selective pressure under antiestrogen therapies.

Activating PIK3CA mutations lead to the activation of downstream oncogenic signaling proteins (Figure 2). PI3K phosphorylates phosphatidylinositol-4,5-bisphosphate (PIP2) into phosphatidylinositol-3,4,5-trisphosphate (PIP3), which recruits oncogenic proteins including serine and threonine kinase Akt [35]. This process can also occur as a result of copy-number loss of the PTEN tumor suppressor gene, which counterbalances the PI3K signaling pathway by de-phosphorylating PIP3 back to PIP2 [36]. When PTEN is lost, downstream effectors of PI3K including Akt become activated without oncogenic signals [37]. Once activated, Akt phosphorylates more oncogenic substrates including the negative regulator proteins of mTOR, TSC2, and PRAS40. mTOR is a common downstream effector of Akt and is responsible for promoting tumor progression and angiogenesis [38].

PI3K/Akt pathway can also be activated through upstream signaling mediated by cell surface receptor tyrosine kinases (RTKs), including the ErbB family, FGFR, EGFR, and other RTKs (Figure 2). ErbB2/HER2 Neu amplification reduces sensitivity to antiestrogen therapy, and this is primarily due to the upstream activation of PI3K/Akt and MAPK signaling pathways leading to estrogen-independent ER phosphorylation and activation [39]. As a result, anti-HER2 therapy is given with antiestrogen therapy when treating HR+ and HER2+ breast cancer.

Furthermore, the PI3K/Akt pathway is downstream from various growth factors including insulin-like growth factor-I (IGF-I) and heregulin. These growth factors utilize the PI3K pathway to activate Erα, leading to estrogen-independent growth [40].

In summary, PI3K pathway activation was shown to promote acquired resistance to long-term endocrine deprivation in preclinical models of ER+ breast cancer cell lines, and proteomic profiling revealed increased phosphorylation of mTOR and Akt [41,42]. This finding revealed that adaptation of HR+ breast cancer cells to estrogen deprivation can lead to tumor dependency on PI3K signaling [42], and both preclinical and clinical data support the efficacy of combination therapies targeting ER and PI3K/Akt pathway to overcome acquired endocrine resistance [41,42]. The SOLAR-1 trial demonstrated that, in PIK3CA-mutant tumors, the addition of the PI3K inhibitor alpelisib to fulvestrant increased PFS from 5.7 to 11 months compared to fulvestrant monotherapy [43].

#### 2.2.3. Mitogen-Activated Protein Kinase (MAPK) Pathway Alterations

MAPK signaling pathway activation promotes cancer proliferation, invasion, and angiogenesis. While KRAS is mutated in only 4% of breast cancer cases, the MAPK pathway is activated in approximately 50% of breast cancers [44,45,46]. In a molecular characterization study of ER+ MBC utilizing circulating tumor DNA (ctDNA), mutations in the NF1 gene were acquired in 8.1% of breast cancers exposed to endocrine therapy [47]. NF1 is a tumor suppressor gene that codes for neurofibromin protein and a key regulatory gene of the MAPK pathway, which negatively regulates RAS by promoting the hydrolysis of RAS-GTP (active form) to RAS-GDP (inactive form). NF1 loss results in constitutively active RAS leading to downstream activation of both PI3K and MAPK pathways (Figure 2) [48]. NF1 loss-of-function mutations are implicated in primary and acquired resistance to ET [31,49]. In a breast cancer cell model harboring loss of NF1 expression, Razavi et al. demonstrated resistance to fulvestrant that was reversed with the addition of an ERK inhibitor [31]. Dysregulated RAS signaling and loss of NF1 can also be associated with ER-independent gene transcriptions including expression of cyclin D1 [48]. Adding CDK4/6 inhibitors to ET in vitro was shown to overcome endocrine resistance driven by NF1 loss [47].

NF1 loss-of-function mutations, RTKs mutations such as ERBB2 activating mutations, as well as alterations in other MAPK pathway genes (EGFR, KRAS, BRAF, and MAP2K1) were found to be enriched in endocrine-resistant advanced ER+ breast cancer compared to early-stage ER+ breast cancer. These alterations were present in 22% of tumors, are associated with a shorter duration of response to subsequent ET, and are mutually exclusive with ESR1 mutations [31]. Inhibitors of RAS and the MAPK pathway oncoproteins (RAF, ERK, and MEK) may be potential therapeutic options to overcome endocrine resistance mediated by MAPK pathway alterations.

#### 2.2.4. Tumor Microenvironment

Beyond the molecularly driven ER-resistant pathways, we have seen the emergence of a new concept linked to the tumor microenvironment (TME) [50]. It is hypothesized that cancer cells do not only interact with their host environment, but also modulate changes that alter all components of their TME including immune cells, vasculature, stromal cells, and extracellular matrix. One such example is related to hypoxia which is a hallmark of worse prognosis in ER-driven tumors [51]. Targeting hypoxia-induced amino acid transporters such as SNAT2 has been postulated to sensitize hypoxic breast cancer cells to antiestrogen treatment [52].

Another example is the inflammatory pathway and metabolic syndrome supporting the association between obesity and increased risk of ER+ breast cancer. Obesity-induced inflammation has been shown to lead to the recruitment of immune cells, specifically macrophages into adipose tissues leading to the activation of proinflammatory transcription factors like NF kappa B, resulting in the development of tamoxifen resistance [53]. However, TME is more complex with the interplay of other factors beyond macrophages, including carcinoma-associated fibroblasts, the extracellular matrix, adipocytes, endothelial cells, and other immune cells [50].

Even though breast cancer, and specifically ER+ breast cancer, is considered an immunologically cold tumor harboring a minimal amount of tumor-infiltrating lymphocytes, a growing body of evidence suggests that the immune host is an active promoter of endocrine-resistant tumors [54]. With that in mind, there are studies investigating TME as a therapeutic target in breast cancer using immunotherapy. The Keynote-028 has demonstrated promising results with the addition of pembrolizumab to chemotherapy in patients with endocrine-resistant metastatic ER+ breast cancer; however, this was a small cohort of 25 patients and more studies are needed. The future remains uncertain, and many attempts are currently underway to potentially transform cold tumors into “hot” with the use of therapeutic vaccines and chimeric antigen receptor (CAR) T cell therapy.

## 3. Current and Novel Therapeutic Approaches

### 3.1. Antiestrogen Therapy

Resistance to tamoxifen due to ESR1 mutations has not been proven outside of in vitro studies, but this agent has an inferior efficacy as a monotherapy for recurrent ER+ MBC, especially in postmenopausal patients [11,55]. While AI were associated with improved outcomes compared to tamoxifen, their use as monotherapy or even in combination with CDK4/6 inhibitors for the treatment of recurrent, advanced ER+ breast cancer is limited by acquired resistance, mainly due to ESR1 mutations. After disease recurrence or progression on prior AI, it is standard practice to switch to fulvestrant +/− CDK4/6 inhibitors or other targeted therapies as indicated [56]. Switching to SERDs (e.g., fulvestrant or oral SERDs when available) after progression on AI is especially important if ESR1 mutation is detected after disease progression.

Novel oral SERDs with improved bioavailability are being developed, and elacestrant is the first FDA-approved oral SERD based on a phase III randomized, EMERALD clinical trial demonstrating significant improvement in PFS with elacestrant when compared to fulvestrant or AI in patients with HR+ MBC, harboring activating ESR1 mutations within the LBD who progressed after CDK4/6 inhibitors and ET [57]. In the EMERALD trial, ESR1 mutations were defined as any missense mutation in codons 310–547 and as defined by Guardant Health for the assay. Elacestrant and other next-generation, orally bioavailable SERDs are under investigation both as monotherapy and in combination with other targeted agents including CDK4/6 inhibitors and PI3K inhibitors, which present promising treatment options to overcome endocrine resistance driven by ESR1 mutations.

### 3.2. CDK4/6 Inhibitors

The introduction of CDK4/6 inhibitors has revolutionized the treatment of ER+ MBC. Currently approved agents include palbociclib, abemaciclib, and ribociclib. They are approved in combination with endocrine therapy for the treatment of ER+ MBC in the first-line setting [58,59,60]. The addition of CDK4/6 inhibitors to ET led to significant improvement in PFS and overall survival [61,62,63,64,65].

Theoretically, ESR1 mutations alone are not expected to cause resistance to CDK4/6 inhibitors since ER is upstream from Cyclin D-CDK4/6 complex-driven cell cycle progression pathway [66]. While the combination of CDK4/6 inhibitors and AI is the most effective, ESR1 mutations can render the AI component ineffective. Furthermore, compared to palbociclib and ribociclib, abemaciclib is the only CDK4/6 inhibitor proven effective as monotherapy in later-line settings, and ESR1 mutations do not lead to resistance to its combination with AI [11,67,68,69].

While ESR1 mutations alone do not lead to resistance to CDK4/6i monotherapy or when combined with fulvestrant, endocrine resistance driven by other mechanisms can potentially create resistance to CDK4/6 inhibitors. Discussion of the resistance mechanism to CDK4/6 inhibitors is beyond the scope of this review.

### 3.3. Everolimus

Everolimus is an inhibitor of mTOR and is approved in combination with exemestane for the treatment of ER+ MBC based on the results of the phase III trial, BOLERO-2 that randomized patients whose disease was refractory to steroidal AI to exemestane with or without everolimus [70]. Adding everolimus was associated with significant improvement in PFS [71]. As discussed above, one endocrine resistance mechanism through which the PI3K/Akt pathway is implicated is ligand-independent ER activation. More specifically, S6 Kinase, which is a substrate of mTOR complex 1 can phosphorylate the activation function domain 1 (AF1) of ERα [72,73]. Everolimus binds to and allosterically inhibits mTORC1, thus overcoming this resistance mechanism [74]. The BOLERO-2 trial was designed before the current understanding of the role of ESR1 mutations in acquired endocrine resistance in patients progressing on prior AI. Thus, the impact of ESR1 mutation and potential resistance to everolimus cannot be concluded. Furthermore, fulvestrant may be a more appropriate antiestrogen partner with everolimus in patients whose disease progresses after AI, and the National Comprehensive Cancer Network guidelines list fulvestrant or tamoxifen as additional ET partner options with everolimus [56].

### 3.4. Alpelisib

PIK3CA gene mutations are identified in approximately 40% of ER+, HER2-negative breast cancer [10,75]. Alpelisib is a PI3K inhibitor that is FDA approved in combination with fulvestrant for the treatment of PIK3CA mutated ER+ MBC [75,76]. In the phase III randomized clinical trial, SOLAR-1 demonstrated improved PFS by adding alpelisib to fulvestrant in patients with PIK3CA mutations [43]. In the SOLAR-1 study, patients were excluded if they had received prior PI3K, Akt, or mTOR inhibitors, and patients had no prior treatment with CDK4/6 inhibitors [43].

Since everolimus inhibits the PI3K/Akt/mTOR cascade downstream of PI3K, its antitumor activity should be independent of PIK3CA mutational status. On the other hand, alpelisib selectively inhibits the growth of ER+ breast tumors harboring PIK3CA mutations making it an ideal agent for PIK3CA mutated breast tumors when compared to everolimus. The synergistic mechanism of combining targets of the PI3K/Akt/mTOR pathway and antiestrogen therapy can be explained by the significant crosstalk between the PI3K/Akt/mTOR pathway and ER [77]. The positive clinical outcomes of adding inhibitors of the PI3K/Akt/mTOR pathway demonstrate that these agents including everolimus and alpelisib can restore hormone sensitivity in breast cancer.

### 3.5. Novel Therapies

Despite recent advances in the treatment of HR+ MBC, this disease remains incurable. Overall survival remains at an average of 2–5 years begging for newer therapies and more innovative strategies [78]. The goal of developing novel ways of therapeutic endocrine manipulations is to delay the use of toxic chemotherapy, improve quality of life, and potentially extend life. We are currently witnessing the emergence of several new therapies designed to overcome endocrine resistance (Table 1).

Newer SERDs such as Camizestrant, Giredestrant, and Imlunestrant, among many others, are currently being investigated extensively in both the metastatic and early-stage settings [79,80]. Newer SERMS such as Lasoxifene is also making it into the clinical arena [81]. Dual PI3K/mTOR inhibitors such as XIN-10 are also being developed and could present a novel therapeutic option to overcome secondary endocrine resistance [82]. Other ways of targeting the estrogen pathway are also being investigated, notably the Proteolysis Targeting Chimera (PROTAC), Selective Estrogen Receptor Covalent Antagonist (SERCA), Complete Estrogen Receptor Antagonist (CERAN) as well as the selective human ER partial agonist (ShERPa). PROTACs facilitate the interaction between ER and ER3 ligase complex causing ER ubiquitination for proteasomal degradation. SERCAs, on the other hand, bind the ER LBD C530 residue, which decreases ER-regulated gene transcription. CERANs bind ER causing its degradation and blocking its complete transcriptional activity. Its unique ability to inactivate both AF1 and activation function 2 (AF2) makes it a promising approach to endocrine-resistant breast tumors [83,84]. ShERPAs are another group of drugs showing promising results specifically in tamoxifen-resistant breast cancer cells [85].

Beyond the estrogen pathway, antiprogestins including selective PR antagonists, are another promising pharmacologic class that can overcome endocrine resistance. This pathway is particularly relevant in ESR1 mutated tumors. There are 3 types of PR antagonists. Type I are complete antagonists that prevent PR dimerization. Onapristone is currently being investigated as monotherapy or in combination with fulvestrant after progression on AI and CDK 4/6 inhibitor [86]. Type II PR antagonists are selective PR modulators like Mifepristone and telapristone. Type III are pure antagonists like Lonaprisan. Both types II and III PR antagonists are still being investigated [87].

The Notch axis also plays an important role. Notch activation can lead to dysregulation of ERα, leading to ERα-dependent transcription in the absence of estrogen, evading estrogen deprivation (Figure 2). Notch inhibition can present another way of overcoming endocrine resistance. Additionally, Aurora Kinase A (AURKA) overactivation has been linked to dysregulation and specifically downregulation of ERα leading to endocrine resistance and worse prognosis. New drugs targeting AURKA, such as Alisertib, which is a highly selective AURKA inhibitor, are being investigated as a potential therapeutic approach [88]. Another potential mechanism for overcoming endocrine resistance would be modulating the JAK-STAT signaling pathway by the lymphocyte adaptor protein, LNK, which is an emerging target being investigated in preclinical studies [89]. And, lastly, the most exciting, recent evidence for the AKT inhibitor, Capivasertib demonstrating improvement in PFS in ER+ MBC in the phase III clinical trial, CAPItello-291, and approval of this agent is highly anticipated.

**Table 1:** Novel and investigational agents for endocrine-resistant advanced ER+ breast cancer.

	Mechanism of Action	Investigational Agents
Oral SERDs [80,90,91,92]	Selective ER degraders	Camizestrant Imlunestrant Giredestrant
Novel SERMs [69,81,93]	Selective ER modulators	Lasofoxifene
SERM/SERD hybrids [94]	SERM/SERD hybrid	Basedoxifene
ShERPA [95,96]	Selective human ER partial agonist	TT-352 BMI-135
PROTACs [97]	Proteolysis targeting chimera ER protein degrader	Vepdegestrant (ARV-471)
CERANs [83,84,98]	Complete estrogen receptor antagonist	Palazestrant (OP-1250)
ShERPA [68,99,100]	Selective estrogen receptor covalent antagonist	H3B-6545
Antiprogestin [101]	Type I antiprogestin	Onapristone
AKT inhibitor [79,102]	Pan Akt kinase inhibitor	Capivasertib
ERK (MAPK) inhibitor [103,104]	Selective, ATP-competitive inhibitor of p38 MAPK α/β	Ralimetinib
FGFR inhibitor [105]	FGFR1–4 inhibitor	Futibatinib
Notch inhibitor [106]	Protein–protein interaction inhibitor that interferes with Notch transcription and signaling	CB-103
AURKA inhibitor [107]	Selective Aurora A kinase inhibitor inducing apoptosis and autophagy	Alisertib

Abbreviations: AURKA, aurora kinase A; AKT, Serine/Threonine Kinase 1; CERAN, Complete Estrogen Receptor Antagonist; ERK, extracellular signal-regulated kinase; FGFR, fibroblast growth factor receptor; MAPK, mitogen-activated protein kinase; PROTAC, Proteolysis Targeting Chimera; SERD, selective estrogen receptor downregulators; SERM, selective estrogen receptor modulators; ShERPA, selective human estrogen receptor (ER) partial agonist.

## 4. Discussion

Over the past few years, more than 10 anticancer drugs have been approved for the treatment of ER+ breast cancer. In endocrine-driven cancers, cytotoxic chemotherapy is typically used after the exhaustion of treatments targeting the estrogen pathway due to toxicity and limited efficacy. Recent FDA approvals have left some questions unanswered, such as the ideal combinations and sequencing of these new agents.

However, the era of precision oncology has ushered in new avenues for personalized care. More studies are delving into genomic-driven treatment selection [108]. The exploration can go beyond single gene alterations, such as the example of mutational signatures [109]. Since genomic alterations in tumor evolution do not happen as single isolated events, understanding the co-occurrence of these events will be critical for appropriate clinical application [110]. As we improve the understanding of how these genomic alterations impact tumor evolution and resistance, and identify specific scenarios where clinical utility exists, the role of repeated ctDNA testing will become evident for treatment de-escalation strategies. Future therapeutic approaches may allow us to de-escalate therapy in select patients while improving effectiveness [111].

In the ER+ metastatic setting, the use of qualitative ctDNA has different potential applications. While there is a clear benefit from identifying mechanisms of resistance to guide treatment selection (e.g., activating ESR1 mutations), it is yet unclear whether early detection of these mutations before radiological progression is associated with clinical benefit. Clinical trials evaluating this concept for metastatic ER+ breast cancer are ongoing (NCT04964934). Moreover, there are ongoing studies investigating the utility of using quantitative ctDNA monitoring to guide dose adjustments and drug holidays, with the ultimate goal of delaying the emergence of resistant strains [112,113].

As we continue to identify new tumor vulnerability and targetable genomic alterations, the value of utilizing comprehensive (i.e., multi-gene or exome sequencing) tumor profiling in the clinical setting will continue to increase. With an optimistic perspective that drug development will catch up with genomic discoveries, repeated ctDNA testing after tumor progression, especially in the form of liquid biopsies, will become critical. By combining biomarker-driven therapies, with a better understanding of resistance mechanisms, and advanced technology that allows us to monitor tumor evolution non-invasively, we must make progress in using highly effective therapies while sparing most patients from the medical and financial toxicity of overtreatment.
